# Occurrence and characterization of *Salmonella* isolates from commercial eggs in Phayao Province, Thailand

**DOI:** 10.14202/vetworld.2025.705-714

**Published:** 2025-03-23

**Authors:** Watsawan Prapasawat, Anchalee Rawangkan, Chittakun Suwancharoen, Atchariya Yosboonruang, Anong Kiddee, Watchara Laenoi, Sirikarn Wiriyasirivaj, Orasa Suthienkul, Achiraya Siriphap

**Affiliations:** 1 Department of Clinic, Faculty of Veterinary Medicine, Mahanakorn University of Technology, Bangkok, 10530, Thailand; 2 Division of Microbiology and Parasitology, School of Medical Sciences, University of Phayao, Phayao, 56000, Thailand; 3 Unit of Excellence on Research and Application of Natural Products for Health and Well-Being, University of Phayao, Phayao, 56000, Thailand; 4 Division of Animal Science, School of Agriculture and Natural Resources, University of Phayao, Phayao, 56000, Thailand; 5 Department of Microbiology, Faculty of Public Health, Mahidol University, Bangkok, 10400, Thailand

**Keywords:** antimicrobial resistance, eggs, food safety, multilocus sequence typing, *Salmonella*, virulence genes

## Abstract

**Background and Aim:**

*Salmonella* contamination in eggs poses a significant public health risk, particularly in alternative egg production systems where contamination and antimicrobial resistance remain underexplored. This study aimed to determine the occurrence of *Salmonella* contamination in three different egg production systems in Phayao, Thailand, and analyze serovar diversity, antimicrobial resistance, virulence genes, and genetic profiles.

**Materials and Methods:**

A total of 750 eggs were sampled from cage, free-range, and organic egg production systems, purchased from supermarkets in Phayao Province. Eggshells and contents were separately analyzed using conventional microbiological methods to isolate *Salmonella*. Phenotypic identification, serotyping, and antimicrobial susceptibility testing were performed. Genotypic characterization, including virulence and antimicrobial resistance gene detection, was conducted using polymerase chain reaction. Multilocus sequence typing (MLST) was employed to determine genetic diversity.

**Results:**

*Salmonella* contamination was detected in three eggshell samples (0.4%), with one positive sample from each production system. The identified serovars were *Salmonella* Mbandaka (cage eggs), *Salmonella* Corvallis (free-range eggs), and *Salmonella* Cerro (organic eggs). Antimicrobial resistance was observed in only one isolate, *S*. Mbandaka, which exhibited resistance to sulfamethoxazole/trimethoprim and carried the *sul1* and *sul2* genes. All *Salmonella* isolates harbored virulence genes (*invA*, *sopB*, and *stn*). MLST analysis identified three distinct sequence types (ST413, ST1541, and ST1593) corresponding to the detected serovars.

**Conclusion:**

This study demonstrates a low occurrence of *Salmonella* contamination in eggshells across different production systems, with no contamination detected in egg contents. The presence of distinct serovars and genetic types suggests varying contamination sources. Although antimicrobial resistance was minimal, the presence of virulence genes in all isolates highlights the potential risk of infection. Continuous monitoring and improved biosecurity measures in egg production and distribution are recommended to enhance food safety and public health.

## INTRODUCTION

*Salmonella* is a major cause of foodborne illness worldwide [1]. The bacteria contaminate foods of animal origin, such as beef, pork, poultry meat, and eggs [2–5]. In Thailand, raw poultry meat is a significant source of non-typhoidal *Salmonella* (NTS), particularly *Salmonella* Enteritidis [1, 6]. Recent findings also indicate that raw or undercooked eggs are important sources of *S*. Enteritidis which contributes to salmonellosis outbreaks [7]. *S*. Enteritidis serovar is primarily associated with foodborne diseases originating from eggs and egg products [8]. In Thailand, raw or undercooked eggs contributed 2.53% of NTS cases [9]. However, eggs can be contaminated with *Salmonella* through fecal contamination of eggshells and transovarial transmission from infected chickens [10, 11]. The egg industry has implemented safety measures and reduced *Salmonella* contamination through its good manufacturing practices, hazard analysis, and critical control points systems [12]. In addition, industrial egg production systems have shifted from conventional caged systems to alternative methods such as free-range and organic systems to support animal welfare and improve egg quality [12]. At present, little is known regarding the prevalence and serovars of *Salmonella* contamination in hen eggs in both conventional and alternative egg production systems.

Moreover, egg contamination with *Salmonella* can cause septicemia and mortality in humans [6]. The severity of infection depends on the expression of virulence genes such as *invA*, *sopB* and *stn* genes [13, 14]. The *invA* and s*opB* genes assist *Salmonella* in interacting with the host cell, facilitating the recognition and invasion of the epithelial cells of the intestinal mucosa [13, 15]. In addition, *stn* gene encodes for enterotoxin production, leading to diarrhea [14]. Furthermore, the use of antimicrobial drugs in the poultry and livestock production industry to treat and prevent bacterial infectious diseases, as well as for growth promotion, have contributed to the growing problem of antimicrobial resistance [16], which become a severe public health issue worldwide [17]. Merati and Boudra [18] have documented the emergence of antimicrobial-resistant bacteria isolated from poultry products. Therefore, the egg production system industry has attempted to decrease antimicrobial use by producing alternative eggs (organic eggs) for consumers. However, there are global studies on *Salmonella* contamination in eggs, and limited data are available for specific production systems in Southeast Asia, including Thailand. Currently, molecular typing techniques are widely used to conduct epidemiological investigations and identify the main sources of infections or outbreaks, which is important for improving public health [19, 20]. Furthermore, the reproducibility of multilocus sequence typing (MLST) is excellent, and the results of sequence types (ST) can be easily shared and compared electronically between laboratories [19].

Despite global efforts to ensure food safety, *Salmonella* contamination in eggs remains a significant public health concern, particularly in alternative egg production systems. While previous studies have extensively documented *Salmonella* contamination in conventional cage eggs, there is limited research on the prevalence, serovar distribution, antimicrobial resistance, and genetic diversity of *Salmonella* in eggs from alternative production systems, such as free-range and organic eggs. The shift from conventional cage systems to alternative methods, driven by consumer preferences and welfare considerations, raises concerns about microbial contamination risks and the effectiveness of current safety measures in these systems. In addition, the relationship between *Salmonella* contamination, antimicrobial resistance, and the presence of virulence genes in different egg production systems remains largely unexplored.

To address this knowledge gap, this study aimed to determine the occurrence of *Salmonella* contamination in eggshells and egg contents from three different egg production systems – cage, free-range, and organic eggs – purchased from super-markets in Phayao, Thailand. Furthermore, this study sought to identify the *Salmonella* serovars present in these eggs, assess their antimicrobial resistance profiles, detect key virulence genes, and determine their genetic diversity using MLST. By providing a comprehensive characterization of *Salmonella* isolates, this study contributes to the understanding of microbial risks in eggs from different production systems and informs strategies for improving food safety and public health.

## MATERIALS AND METHODS

### Ethical approval

This research did not involve human or animal subjects, hence ethical approval was deemed exempt for this study.

### Study period and location

This study was conducted from January to December 2019. The three types of commercial egg samples in this study were purchased from three supermarkets located in Phayao Province, Thailand. The samples were processed at Microbiology Laboratory, School of Medical Sciences, University of Phayao.

### Sample collection

In this study, three types of egg production systems were included: (1) Cage eggs (hens living in intensive production housing systems), (2) free-range eggs (hens reared in free-run (barn or aviary) housing systems, with access to outdoor runs), and (3) organic eggs (hens raised in free-range housing systems and only fed organic certified feed). A total of 750 commercial egg samples were purchased from three supermarkets located in Phayao Province, Thailand in 2019. The sample unit was a single egg. From each supermarket, randomly selected 250 samples (83–84 eggs per type) from stratified random sampling, with their certificate labels to confirm their production system, as indicated on the packages. The samples were then kept in an icebox and immediately transferred to the Microbiology Laboratory at the School of Medical Sciences, University of Phayao, for further isolation of *Salmonella*.

### Isolation and identification of *Salmonella*

The bacteria on the eggshells were collected by soaking the egg in 10 mL of buffered peptone water (BPW; Oxoid, Basingstoke, UK) in a sterile plastic bag for 10 min. The egg was then removed from the BPW and decontaminated by soaking in 70% ethanol for 10 min. The eggshells were then removed, and the contents were aseptically collected and transferred to 225 mL of BPW. The BPW samples were incubated at 37°C for 18–24 h and transferred to selective enrichment Rappaport-Vassiliadis (RV) broth (Difco, BD, Detroit, MI, USA) and incubated at 42°C. After 18–24 h incubation, RV broth cultures were inoculated onto xylose lysine deoxycholate agar (Difco, BD) and incubated at 37°C for 18–24 h. Suspected colonies (a black center and slightly red translucent zone) or hydrogen sulfide-negative *Salmonella* (e.g., *Salmonella* Paratyphi A) were picked from each individual sample for further biochemical identification using the triple sugar iron (Oxoid) and lysine-indole motile (Oxoid) tests [21]. All *Salmonella* strains with positive test results were identified as *Salmonella* and were preserved as stocks in 20% glycerol and stored at –20°C until further use.

### *Salmonella* serotyping

The identified *Salmonella* strains were further sero-grouped with commercial polyvalent O antisera (S&A Reagents Lab, Bangkok, Thailand) through slide agglutination according to the Kauffman-White Scheme [22]. Each positive serogroup of *Salmonella* was further identified serovar and submitted to the World Health Organization (WHO) National *Salmonella* and *Shigella* Reference Center Laboratory, Department of Medical Science, Ministry of Public Health, Nonthaburi, Thailand.

### DNA extraction

The *Salmonella* strains were sub-cultured in Luria-Bertani broth (LB Broth; Difco, Detroit, MI, USA) and incubated at 37°C for 15−18 h, and then their DNA was extracted using Qiagen’s QIAamp^®^ DNA mini kit (Qiagen, Hilden, Germany) according to the manufacturer’s protocols. The extracted DNA of *Salmonella* was stored at −20°C until use.

### Antimicrobial susceptibility testing

The agar disk diffusion method was performed according to the guidelines of the Clinical and Laboratory Standards Institute [23]. All *Salmonella* strains were tested for 15 antimicrobial agents, including gentamicin, kanamycin, streptomycin, chloramphenicol, imipenem, meropenem, cefotaxime, ceftazidime, cefepime, ampicillin, amoxicillin/clavulanic acid, ciprofloxacin, nalidixic acid, sulfamethoxazole/trimethoprim (SXT), and tetracycline (Oxoid). Mueller-Hinton agar (Oxoid) was used as the culture medium for the test. *Escherichia coli* American Type Culture Collection 25922 was used as the reference strain for quality control.

### Antimicrobial resistance and virulence genes

All *Salmonella* strains were screened for eight antimicrobial-resistant genes (*tetA*, *tetB*, *blaTEM, blaSHV, sul1*, *sul2*, *aadA*, and *strA/strB*), and three virulence genes (*invA, stn*, and *sopB*) using polymerase chain reaction (PCR) amplification. The primer sequences used in this study are listed in Table 1 [24–28]. PCR was performed using the manufacturer’s protocol followed by Bio-Helix (Taiwan). The amplicons were analyzed by 1.5% agarose gel electrophoresis, stained with ethidium bromide, and visualized under ultraviolet light using a gel documenting system (BIS 303 PC, Jerusalem, Israel). *Salmonella* Typhimurium Thailand Institute of Scientific and Technological Research 1469 and *E*. *coli* TEC110 (unpublished) were used as positive controls.

**Table 1:** Oligonucleotide primers used for PCR amplification in this study.

Target gene	Primer	Sequence (5’➔3’)	Annealing temperature (°C)	Amplicon size (bp)	References
*tetA*	*tetAF*	GCTACATCCTGCTTGCCTTC	55	210	[24]
	*tetAR*	CATAGATCGCCGTGAAGAGG			
*tetB*	*tetBF*	TTGGTTAGGGGCAAGTTTTG	55	659	[24]
	*tetBR*	GTAATGGGCCAATAACACCG			
*blaTEM*	*TEMF*	ATTCTTGAAGACGAAAGGGC	60	1150	[24]
	*TEMR*	ACGCTCAGTGGAACGAAAAC			
*blaSHV*	*SHVF*	CACTCAAGGATGTATTGTG	60	885	[24]
	*SHVR*	TTAGCGTTGCCAGTGCTCG			
*sul1*	*sul1F*	CTTCGATGAGAGCCGGCGGC	68	417	[24]
	*sul1R*	GCAAGGCGGAAACCCGCGCC			
*sul2*	*sul2F*	AGGGGGCAGATGTGATCGAC	58	249	[24]
	*sul2R*	GCAGATTTCGCCAATTG			
*aadA*	*4F*	GTGGATGGCGGCCTGAAGCC	63	525	[25]
	*4R*	AATGCCCAGTCGGCAGCG			
*strA/strB*	*strAF*	ATGGTGGACCCTAAAACTCT	63	893	[26]
	*strBR*	CGTCTAGGATCGAGACAAAG			
*invA*	*invA-F*	GTGAAATTATCGCCACGTTCGGGCAA	64	284	[27]
	*invA-R*	TCATCGCACCGTCAAAGGAACC			
*stn*	*stn-F*	CTTTGGTCGTAAAATAAGGCG	64	260	[27]
	*stn-R*	TGCCCAAAGCAGAGAGATTC			
*sopB*	*sopB-F*	CAACCGTTCTGGGTAAACAAGAC	64	1378	[27]
	*sopB-R*	AGGATTGAGCTCCTCTGGCGAT			
*hisD*	*hisD-F*	GAAACGTTCCATTCCGCGC	55	894	[28]
	*hisD-R*	GCGGATTCCGGCGACCAG			
*thrA*	*thrA-F*	GTCACGGTGATCGATCCGGT	55	852	[28]
	*thrA-R*	CACGATATTGATATTAGCCCG			
*aroC*	*aroC-F*	CCTGGCACCTCGCGCTATAC	55	826	[28]
	*aroC-R*	CCACACACGGATCGTGGCG			
*purE*	*purE-F*	GACACCTCAAAAGCAGCGT	55	510	[28]
	*purE-R*	AGACGGCGATACCCAGCGG			
*sucA*	*sucA-F*	CGCGCTCAAACAGACCTAC	55	643	[28]
	*sucA-R*	GACGTGGAAAATCGGCGCC			
*hemD*	*hemD-F*	GAAGCGTTAGTGAGCCGTCTGCG	55	666	[28]
	*hemD-R*	ATCAGCGACCTTAATATCTTGCCA			
*dnaN*	*dnaN-F*	ATGAAATTTACCGTTGAACGTGA	55	833	[28]
	*dnaN-R*	AATTTCTCATTCGAGAGGATTGC			

PCR=Polymerase chain reaction

### MLST assay

MLST was performed following the method described by Bell *et al*. [28]. PCR amplification of seven housekeeping genes of *Salmonella* was performed using seven primer pairs targeting *hisD*, *thrA*, *aroC*, *purE*, *sucA*, *hemD*, and *dnaN* (Table 1). The PCR products were sequenced using the Sanger sequencing method at Macrogen Inc. in South Korea. The sequences were analyzed using BioEdit version 7.2 (https://bioedit.software.informer.com/7.2/). The sequences of the seven housekeeping genes were then compared and aligned with the MLST online database (https://pubmlst.org/). Subsequently, the sequences were further submitted to the online *Salmonella* MLST database to obtain the allele numbers and STs (https://enterobase.warwick.ac.uk).

### Statistical analysis

Descriptive statistical analysis was conducted to determine the prevalence of *Salmonella* contamination, serovars, antimicrobial resistance, virulence genes, and STs, expressed in frequencies and percentages. Fisher’s exact test was applied to compare *Salmonella* contamination rates among the three egg production systems, with statistical significance set at p < 0.05.

To assess the agreement between phenotypic antimicrobial resistance and the presence of resistance genes, Cohen’s kappa statistic was employed, with values interpreted as follows: Slight agreement (0.01–0.20), fair agreement (0.21–0.40), moderate agreement (0.41–0.60), substantial agreement (0.61–0.80), and almost perfect agreement (0.81–1.00).

All statistical analyses were performed using the Statistical Package for the Social Sciences (SPSS) software (version 18.0, SPSS Inc., Chicago, IL, USA), with p < 0.05 considered statistically significant.

## RESULTS

### Occurrence of *Salmonella* in eggshells and egg contents

The occurrence of *Salmonella* in eggshell and egg content samples is summarized in Table 2. From 750 eggshell samples, only 3 samples (0.4%) were positive for *Salmonella*. Among the three positively contaminated eggs, one each (0.4%; 1/250) was detected in cage eggs, free-range eggs, and organic eggs (Table 2). There is no statistically significant differences between *Salmonella* contamination and egg production systems (p = 1.00). Serotyping of the *Salmonella* strains corresponded to serovars Mbandaka, Corvallis, and Cerro from cage egg, free-range, and organic egg samples, respectively (Table 2). No *Salmonella* contamination was detected in the egg contents of the three egg production systems.

**Table 2:** Occurrence of *Salmonella* contamination, serovars, and antimicrobial resistance in egg samples from three egg production systems.

Source	No. of examined samples	No. (%) of positive samples		Total (%) of positive samples	Serovar	Antimicrobial resistance phenotype	Antimicrobial resistance genes

		Eggshell	Egg content				
Cage	250	1 (0.4)	0	1 (0.4)	Mbandaka	SXT	*sul1, sul2*
Free range	250	1 (0.4)	0	1 (0.4)	Corvallis	-	-
Organic	250	1 (0.4)	0	1 (0.4)	Cerro	-	-
Total	750	3 (0.4)	0	3 (0.4)	-	-	-

SXT=Sulfamethoxazole/trimethoprim

### Detection of phenotypic and genotypic resistance to *Salmonella* isolates

*Salmonella* strains from three different egg production systems were susceptible to all 15 tested antimicrobial agents, except for *Salmonella* Mbandaka showed phenotypic resistance to SXT (33.3%; 1/3).

Furthermore, antimicrobial resistance genes were also analyzed using PCR. This study revealed that *S*. Mbandaka SXT-resistant strain (33.3%; 1/3) carried *sul1* and *sul2* genes, whereas two phenotypically anti-microbial susceptible strains of *Salmonella* Corvallis and *Salmonella* Cerro did not carry resistance genes (Table 2). These results revealed that there was concordance (Kappa = 1.00) between phenotypic resistance and the presence of antimicrobial resistance genes (100%, 1/1).

### Detection of virulence genes and MLST in *Salmonella* isolates

Regarding the virulence genes, all three serovars (*S*. Mbandaka, *S*. Corvallis, and *S*. Cerro) from three different egg production systems were found to carry *invA* gene (284 bp), *stn* gene (260 bp), and *sopB* gene (1378 bp), as shown in Table 3. The genetic relationships of all *Salmonella* strains from the three different egg production systems were analyzed using MLST. STs and allelic profiles of each *Salmonella* strain are presented in Table 3. Three *Salmonella* serovars were assigned to distinct STs, including *S*. Mbandaka ST413 (from cage egg samples), *S*. Corvallis ST1541 (from free-range eggs), and *S*. Cerro ST1593 (from organic eggs) (Table 3). It was concluded that *S*. Mbandaka ST413 isolated from cage egg samples was SXT-resistant and expressed the *sul 1* and *sul 2* genes.

**Table 3:** Distribution of virulence genes, allele profiles, and STs of *Salmonella* isolated from three egg production systems.

Sources	Serovars	Virulence genes			Allele type							ST
	
		*invA*	*stn*	*sopB*	*aroC*	*dnaN*	*hemD*	*hisD*	*purE*	*sucA*	*thrA*	
Cage	Mbandaka (n = 1)	1	1	1	15	70	93	78	113	6	68	413
Free range	Corvallis (n = 1)	1	1	1	197	187	10	234	8	65	22	1541
Organic	Cerro (n=1)	1	1	1	222	105	46	123	225	115	115	1593

ST=Sequence type

## DISCUSSION

At present, the egg production industry has transitioned from conventional caged systems to alternative systems, such as free-range and organic systems. This shift is due to political, commercial, and social pressures [12] and consumer preferences. Many salmonellosis outbreaks around the world have been linked to eggs and egg products as a common source of infection [29]. Eggs can be contaminated through two routes: Vertical transmission from infected chickens or horizontal transmission through fecal contamination [11]. The vertical transmission is commonly linked to *S*. Enteritidis and *S*. Typhimurium can be controlled by vaccination in breeder and commercial layers [11]. This study reported *Salmonella* contamination in eggshells only, with no contamination in egg contents. *Salmonella* contamination of eggshells was detected in samples from three egg production systems: Cage eggs (0.4%), free-range eggs (0.4%), and organic eggs (0.4%). Interestingly, no significant difference in contamination was observed between the three egg production systems. Solís *et al*. [12] also reported no difference in the prevalence of *Salmonella* contamination in eggs between conventional and alternative production systems. Similarly, Whiley and Ross [30] reported that the low detection rates of *Salmonella* contamination in eggs from caged, barn, and free-range egg productions were not significantly different. The results of this study agreed with the low prevalence of *Salmonella* contamination in eggs in different countries, such as the USA (0.5%, 2/426) [12], China (0.5%, 27/5548) [31], and Iran (0.5%, 3/600) [32]. In contrast, many countries reported higher detection rates, that is, in Algeria, 7.2% (13/180) of *Salmonella* contamination in commercial eggs [18], and 13.8% (61/440) contamination in tested eggs collected from wet markets in China [33]. In Australia, 11.5% (23/200) contamination in retail eggs [34], and in India, 5.6% (17/300) of eggs were contaminated in wholesale and retail markets [35]. The different levels of *Salmonella* detection rates on eggs found in each country might be due to many possibilities: Different regions and countries [36], housing systems, farm management (improper washing, grading, and packing operation), egg storage process, and distributors (fresh market/supermarket) [12, 37–40]. However, in this study, the low level of *Salmonella* contamination in eggs from three types of egg production systems might be related to production processes, and storage conditions in production systems, including supermarkets, showed rather good hygiene in each process. Nevertheless, to ensure food safety and reduce the risk of foodborne diseases, it is crucial to enhance control measures and conduct ongoing surveys at chicken farms, during transportation, at points of sales, and in storage facilities [32]. Although *Salmonella* contamination was found only on eggshells, not in the content, it may be cross-contaminated through egg contents during egg handling by consumers [11, 12]. Therefore, it is important for consumers to clean or rinse eggshells before cooking and to thoroughly wash their hands after handling eggs. This practice helps prevent cross-contamination [41]. Moreover, consumers should avoid raw or undercooked eggs.

Various *Salmonella* serovars, including *S*. Enteritidis and *S*. Typhimurium, are commonly found in eggshells and egg products, which are often associated with food poisoning [8]. In contrast, this study found that *S*. Mbandaka, *S*. Corvallis, and *S*. Cerro were from cage eggs, free-range eggs, and organic eggs, respectively. The results of the present study agreed with previous reports of *Salmonella* serovars, such as *S*. Mbandaka [42], *S*. Cerro [43], and *S*. Corvallis [44], which have also been found at a low frequency on egg surfaces and in food samples. In addition, similar findings have been reported in many studies conducted in various countries, such as, the UK [43], Thailand [44], Sri Lanka [45], Japan [46], and South India [47]. It has also indicated that egg samples can be contaminated by different *Salmonella* serovars [8]. The prevalence of *Salmonella* serovars in egg samples varies according to sample type, sample collection method, and geographic area [8, 44].

Antimicrobial agents are frequently used in the poultry industry for therapeutic, growth promotion, and disease prevention [48]. Our results showed that all *Salmonella* strains were susceptible to most antimicrobial tested (14 agents). In addition, multidrug resistant strains were not detected, which is inconsistent with previous studies by Singh *et al*. [35], Sornplang *et al*. [44], and Utrarachkij *et al*. [49]. The *S*. Mbandaka was found in cage egg samples that were resistant to SXT but not found in *S*. Corvallis from free-range eggs and *S*. Cerro from organic eggs. The prevalence of antimicrobial resistance was found only in cage eggs. Pande *et al*. [50] indicated that specific serovars, like *S*. Mbandaka, are associated with antimicrobial resistance. The variation in resistance among serovars could be attributed to the selective transfer of mobile genetic elements carrying antimicrobial resistance among *Salmonella* serovars [51]. For instance, the presence of a class 1 integron typically carries the *sul1* and *qacE* genes in its conserved region, which confer resistance to sulfamethoxazole and quaternary ammonium compounds [52, 53]. Therefore, the monitoring of integrons is essential for further analysis. In addition, the differences in antimicrobial resistance between cage eggs (cage) and alternative eggs (non-cage) may largely result from variations in antimicrobial use and farming practices between cage and non-cage farms. In addition, environmental factors and human influence from farm to retail should not be overlooked [54]. Furthermore, alternative hen eggs production systems, such as free-range and organic production systems, have grown in popularity to reduce antimicrobial use on farms and address animal welfare [12]. This study also revealed a correlation between phenotypic resistance and antimicrobial resistance genes in *S*. Mbandaka compared with a previous study of non-expressing genes by Pande *et al*. [50]. Although this finding demonstrated a low rate of antimicrobial resistance in *Salmonella* based on egg samples, it is essential to implement measures to control and monitor the use of antimicrobial agents in farms to reduce their antimicrobial resistance.

The virulence factors of bacteria are potential contributors to the ability of *Salmonella* to cause infections [33]. All *Salmonella* strains from the three egg production systems carried all tested virulence genes (*invA*, *stn*, and *sopB*). These virulence genes support the interactions of *Salmonella* with host cells; for example, *invA* and *sopB* genes are involved in host recognition and invasion of the epithelial cells of intestinal mucosa [13, 15]. The *stn* gene encodes for enterotoxin production [14]. This result is consistent with the findings of Zou *et al*. [55], which reported >90% of cases of *Salmonella* *stn* and *sopB* genes. Moreover, Farahani *et al*. [56] reported a prevalence of 100% for *inv*A gene [56]. These findings indicated that these virulence genes are widespread in *Salmonella* [14, 53] and affect the severity of *Salmonella* infection [33]. Furthermore, the presence of certain virulence genes affects human health by contributing to diarrhea and gastroenteritis. This approach also imposes a financial burden on health systems due to the costs associated with infection control, diagnosis, and treatment [57].

Molecular typing methods, such as MLST, can be used for the phylogenetic investigation of *Salmonella* agents [58]. The phenotypic and genotypic characteristics of *Salmonella* with serovars and MLST were identified. The present study demonstrated that three *Salmonella* strains were assigned to 3 distinct STs, namely, ST413 (*S*. Mbandaka), ST1541 (*S*. Corvallis), and ST1593 (*S*. Cerro). This result demonstrates that *S*. Mbandaka ST413 expressed antimicrobial resistance and virulence genes. The dissemination of *S*. Mbandaka ST413 has been detected in poultry farms [59] and humans [60]. This finding is in accordance with a previous study by Benevides *et al*. [53] which reported *S*. Mbandaka ST413 circulating in egg-laying flocks and associated with strong antimicrobial resistance and virulences. *S*. Mbandaka ST413 is genetically close to strains involved in foodborne outbreaks and invasive salmonellosis cases worldwide. *S*. Corvallis ST1541 is typically less common than other *Salmonella* serovars [61]. However, *S*. Corvallis ST1541 has recently emerged as a globally disseminated pathogenic strain that often causes severe foodborne infections in chickens rather than eggs [62]. *S*. Cerro ST1593 has been reported in human clinical and environmental sources [63]. There are many STs of *Salmonella* circulating in poultry farms, egg processing, and egg products. For example, ST11 and ST1925 were found in chicken in Malaysia [64], ST11 was found in poultry farms in Bangladesh [65], ST1954 was found in poultry in Tetouan-Morocco [66], and ST1, ST3, and ST4 were found in eggs in Pennsylvania [67]. The possibilities of the diversity of STs of *Salmonell*a might be related to sample types and countries.

## CONCLUSION

This study comprehensively analyzes *Salmonella* contamination in eggs from different production systems in Phayao, Thailand. The findings indicate a low prevalence of *Salmonella* contamination (0.4%) in eggshells across cage, free-range, and organic eggs, with no contamination detected in egg contents. The identified *Salmonella* serovars (*S*. Mbandaka, *S*. Corvalli*s*, and *S*. Cerr*o*) exhibited distinct STs (ST413, ST1541, and ST1593). While only one strain (*S*. Mbandaka from cage eggs) displayed antimicrobial resistance, all isolates carried virulence genes (*invA, sopB*, and *stn*), indicating potential pathogenicity. These findings highlight the importance of continuous monitoring and biosecurity measures to minimize *Salmonella* contamination and ensure food safety.

The study’s strengths lie in its comprehensive phenotypic and genotypic analysis, which provides a detailed characterization of *Salmonella* isolates. The comparative approach across different egg production systems offers valuable insights into microbial risks, while the use of MLST enhances epidemiological understanding. The research contributes to public health by identifying the presence of virulence and antimicrobial resistance genes in *Salmonella* strains, emphasizing the need for preventive measures in egg production and distribution.

However, certain limitations must be acknowledged. The sample size, while substantial, could be expanded to improve the generalizability of findings. The study is limited to Phayao Province, restricting its applicability to broader geographic regions. In addition, farm-level surveillance was not conducted, preventing a direct assessment of contamination sources. Cross-contamination risks during storage, transportation, and consumer handling were also not evaluated, which are critical factors influencing foodborne transmission.

Future studies should expand sample collection to multiple regions and increase sample size to enhance statistical robustness. Investigating *Salmonella* prevalence at poultry farms would provide deeper insights into contamination pathways. Longitudinal studies could help identify seasonal variations in *Salmonella* prevalence and antimicrobial resistance patterns. Whole-genome sequencing (WGS) could further elucidate genetic mechanisms underlying antimicrobial resistance and virulence factors. Research on consumer handling, storage, and preparation practices would help assess contamination risks at the household level. In addition, comparative studies with other foodborne pathogens in eggs and poultry products would provide a broader perspective on microbial risks in the food supply chain.

This study serves as a critical step in understanding *Salmonella* contamination in commercial eggs, emphasizing the need for continued surveillance, improved management practices, and consumer awareness to mitigate foodborne risks.

## AUTHORS’ CONTRIBUTIONS

WP: Designed the study, performed data analysis, and drafted and revised the manuscript. AR and CS: Designed the study, collected the samples, and performed bacterial isolation. AY and AK: PCR and molecular detection. WL and SW: MLST interpretation and analysis. OS: Designed the study, interpreted the results, and drafted and revised the manuscript. AS: Designed the study, data analysis, and revised the manuscript. All authors have read and approved the final manuscript.
